# Policy Analysis on Recycling of Solid Waste Resources in China—Content Analysis Method of CNKI Literature Based on NVivo

**DOI:** 10.3390/ijerph19137919

**Published:** 2022-06-28

**Authors:** Junjie Li, Xuehang Sun, Xin Dai, Junying Zhang, Bangfan Liu

**Affiliations:** 1 School of Public Administration, Yanshan University, Qinhuangdao 066004, China; m15033502089@163.com (J.L.); zhangjunying0809@163.com (J.Z.); 2 Hebei Public Policy Evaluation and Research Center, Qinhuangdao 066004, China; daixin860227@163.com; 3 Institute of Marxism Chinese Academy of Social Sciences, Beijing 300712, China

**Keywords:** solid waste resources, recycling, policy analysis, NVivo, content analysis method, CNKI

## Abstract

The recycling of solid waste resources can effectively alleviate resource shortages and environmental pollution and can promote the sustainable development of an ecological economy and green economy. Therefore, China has set up a series of recycling policies. In order to promote the development of China’s solid waste resource recycling industry, and to be able to set up and implement correct policy documents according to real-time dynamics, this study uses NVivo software to analyze the content of 10 Core Journals’ documents screened from the China HowNet database and sets up three node classifications and four partition dimensions to build a three-dimensional model for qualitative analysis and emotional analysis. The analysis determines the existing weaknesses of China’s solid waste resource recycling policy under four dimensions and puts forward prospects for its future from these four aspects of system, capacity, measures and environment.

## 1. Introduction

With the development of social economy, people’s living standards have been continuously improved; however, considering the vigorous development of industrialization and urbanization, the extensive economic development model has caused serious social problems [1]. The generation of solid waste in China involves almost all industries of the national economy. In the process of separation, collection, transfer, treatment, and recycling of solid waste, if the safety management cannot be realized, it will not only waste recyclable resources, but also easily cause serious environmental pollution, which will endanger the health of citizens and social development [2,3,4]. Some Chinese scholars have also made many corresponding explanations on the adverse effects of the extensive economic development model. For example, the following three authors made similar expressions. Zhu Dongbo and Ren Li, etc. (2018): “Since the reform and opening up policy was adopted, China has experienced a booming economic growth, which provides a important material basis for the development. However, the non effective development of economy has led to a serious environmental concern” [5]. Liang Longwu and Wang Zhenbo, etc. (2019): “In the rapid urbanization process of the Beijing-Tianjin-Hebei urban agglomeration, the extensive economic development model of“high energy consumption, high pollution, and high emissions”has caused a series of problems such as smog water pollution, and ecological system destruction“ [6]. Liu Ruiwen (2022): “In China, the problem of transboundary water pollution in basin has attracted much attention. To solve it, a series of policies have been initiated. However, the enforcement of these policies actually is faced with the difficulty of being blocked due to the constraints of water mobility, territorial fragmentation management, “ extensive” economic development model, “ local protectionism”, and lack of inter-governmental cooperation” [7]. According to the data, Article 3 of the general provisions of the law of the people’s Republic of China on the prevention and control of environmental pollution by solid waste promulgated by order No. 31 of the president of the people’s Republic of China, “the State shall prevent and control environmental pollution by solid waste, implement the principles of reducing the generation and harmfulness of solid waste, making full and rational use of solid waste and harmless disposal of solid waste, and promote the development of cleaner production and circular economy.” Therefore, solid waste recycling is one of the best ways to solve the problem of resource shortage, the dilemma of waste and garbage, and the development of circular economy and sustainable economy. Therefore, the research on China’s solid waste resource recycling policy can not only help to set up and implement correct policy documents according to real-time dynamics, but also promote the development of China’s solid waste resource recycling industry and is of great significance to the construction of socialism with Chinese characteristics and the great rejuvenation of the Chinese nation. At the same time, it is found that there is no qualitative research on the recycling policy of solid waste resources in China. Therefore, the author uses NVivo software to conduct this research, which will fill the gap in this field, so as to achieve the goal of helping policy research and industrial development in this field.

## 2. Materials and Methods

Content analysis method is a quantitative analysis method based on qualitative problem assumptions and uses statistical analysis methods and tools to process research samples and draw qualitative conclusions from statistical data. The content analysis method was initially applied in the field of communication and began to rise in various fields in recent years. With the help of NVivo software, this paper carries out text coding, and studies the recycling policy of solid waste resources in China through the visual presentation of coding.

### 2.1. Literature Collection

This paper began to collect relevant data and review literature in April 2022. The data collection is based on Chinese academic journals of CNKI. The data source categories are uniformly selected as a core of Peking University and CSSCI. The retrieval conditions: the theme is “recycling of solid waste resources”; the matching method is selected as accurate; check synonym expansion. In order to prevent the contents of the master’s thesis and conference from duplicating with the contents of periodicals, master’s thesis and conference thesis, as well as some literature such as book review, conference, solicitation of contributions, and news irrelevant to the research topic, were eliminated. A total of 10 relevant journal literatures were selected, and the time span was 2005–2021 [8,9,10,11,12,13,14,15,16,17].

### 2.2. Research Tool

In this paper, NVivo 11 qualitative analysis software is used as the coding tool. The PDF versions of 10 journal documents exported from HowNet (CNKI) are simply sorted and imported into the software as internal materials for coding and analysis. As a data analysis tool of the latest version of specialized qualitative data, NVivo is mainly used to process non-quantitative and unstructured qualitative data and outputs the results in quantitative form. Therefore, NVivo has formed a quantitative paradigm of literature research so as to realize universality, objectivity, and a visualization similar to that in big data analysis.

### 2.3. Analysis of Basic Literature

**Figure 1:**
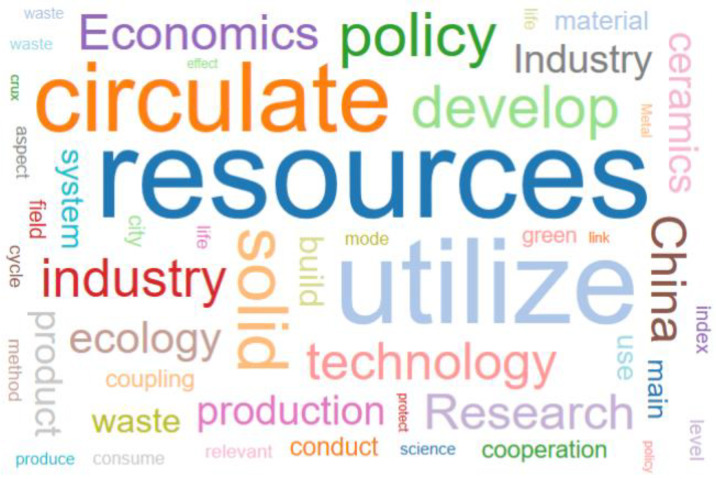
High-frequency word cloud.

**Figure 2:**
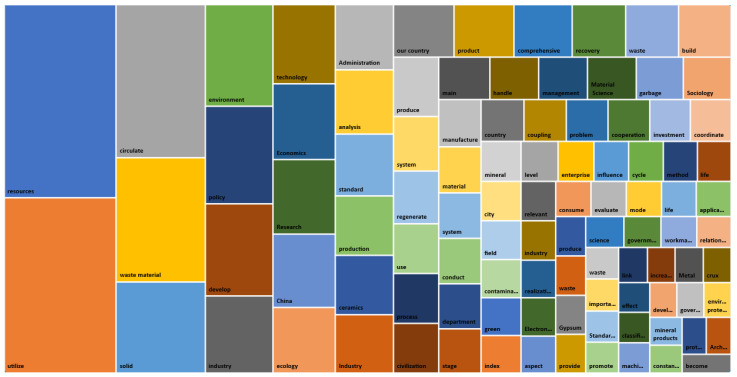
Tree structure of high word-frequency moment form.

We queried the word frequency in ten documents through Nvivo software and set the words displayed to 100. The minimum length was set to 2. We set groupings as synonyms. After running the query, we added some inappropriate words to the stop word list and exported the formed word cloud and moment tree structure, as shown in Figure 1 and Figure 2. Among all the statistical word frequencies, “utilization”, “resources”, “waste”, “recycling”, “solid”, “development”, “environment”, “policy”, “production”, and “management” were the ten groups with the highest statistical amount. On the one hand, this verifies the correctness of the selected analysis text, and on the other hand, it also shows that research into the recycling policy of solid waste resources in China mainly focuses on “development”, “environment”, “production”, “management”, and other aspects.

### 2.4. Establishment and Composition of Analysis Dimension

**Figure 3:**
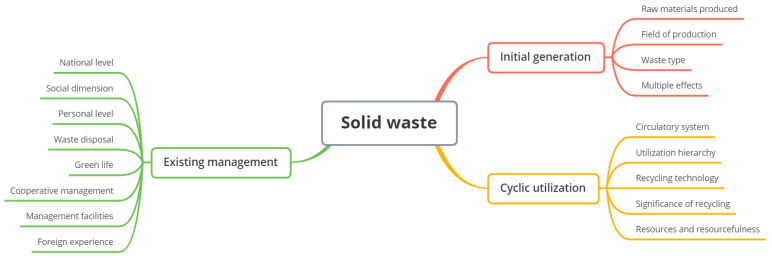
Mind map of literature research frameworks.

First, we encode the imported data into NVivo’s internal materials. Secondly, according to the program rooted theory, NVivo software is used to encode the document content, and the software automatic coding and manual coding functions are used to encode. The coding mainly uses descriptive language. Third, classify and summarize all source nodes to form 2257 open codes, 17 spindle codes, and 3 selective codes for initial generation, existing management, and recycling. The generation of selective codes is based on the outgoing process of solid waste resources. We combined the nodes of the three levels and drew the mind map with x-mind software (see Figure 3).

On the basis of the above, the four dimensions of this study—industrial field, technical support, policy attribute, and circulation background are determined. The “industrial field” dimension corresponds to the initial generation of solid waste; “technical characteristics” and “policy attributes” dimensions correspond to the two levels of solid waste disposal management; “recycling background” dimension corresponds to the part of solid waste entering the recycling application. In the case node classification, the classification cases of four dimensions are constructed, and the actual situation of each dimension is analyzed by constructing the node matrix code.

The composition and analysis of codes under all levels of dimensions were as follows:

First is the literature research under the dimensions of different industries and fields [18,19]. Through coding analysis of the literature content, this paper summarizes the direct or indirect expression of the literature in this regard, analyzes the research situation regarding China’s solid waste resource recycling policy in different industries from three aspects, industrial fields, other fields, and mixed fields, and takes these three aspects as the attribute values of the case node classification of NVivo software analysis.

Second is the literature research under different technical feature dimensions [20,21,22]. Through the analysis of the literature content, it is found that most researchers use technical means in the process of solid waste recycling for further research, and the technology is integrated into the field of problem generation. Therefore, this paper studies this from three aspects: industrial production technology, waste treatment technology, and domestic production technology, and lists it as the attribute value used for case node classification in NVivo software.

Third is the literature research under different policy attribute dimensions [23,24]. From the retrieved literature, the policies adopted by Chinese policymakers regarding the recycling of solid waste are mainly divided into environmental protection policies and economic policies. Therefore, this paper discusses and analyzes the application of environmental protection and economic policies. This is applied as the attribute value in the case node classification.

Fourth is the literature research under different circulation background dimensions [25,26,27]. Based on the grounded theory, this study aims to analyze the background conditions of the generation and application of solid waste recycling policy, explore the driving force behind them, and then provide a basic direction for follow-up research. At the same time, this is used as the attribute value of the case classification node for matrix coding analysis.

## 3. Document Matrix Coding Analysis

NVivo software is used to encode the contents of documents, forming a coding system with initial generation, existing management and recycling as tree nodes, and the original information of documents as free nodes. Through careful study of the literature content and coding information, a three-dimensional analysis model is formed from the four dimensions of industry field, technical characteristics, policy attributes, and circular background, and then the research results are qualitatively analyzed. Its purpose is to understand the qualitative factors of the selected literature in different dimensions, so as to form the data results of text content analysis and provide a data basis for future prospects.

### 3.1. Literature Research and Analysis under the Dimension of Industry Field

**Figure 4:**
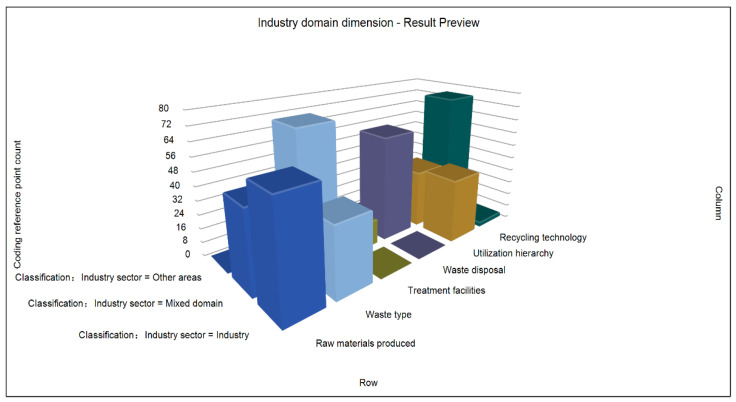
Counting diagram of matrix coding reference points under industry domain dimension.

In this dimension, according to the analysis of the literature, the secondary nodes of “raw materials”, “waste type”, “treatment facilities”, “waste disposal”, “utilization level”, and “recycling technology” of China’s solid waste resources are selected from the three primary nodes of initial generation, existing management, and recycling for analysis. In addition, the matrix coding reference point counting chart is generated (see Figure 4). It can clearly be seen from the 3D column chart that, in terms of the mixing field, the industrial field, and other fields, the research on solid waste resources mainly focuses on the mixing field, followed by the industrial field. This shows that there is a distribution of emphases. Though it pays enough attention to the industrial industry, it does not provide good consideration to other fields. As experts and scholars put it, “green ecological design and cleaner production must be carried out in the industrial production and manufacturing link; green supply chain must be built in the circulation and consumption link and green consumption lifestyle must be advocated; new business models must be explored in the recycling and disposal link and scientific and technological innovation of solid waste recycling in key fields and varieties must be increased” [8]. At the same time, it can be seen from the figure that the industry inputs the rawest materials; the waste type is mainly comprised of products in the mixed field. In terms of treatment facilities, they are at a low level as a whole. The level of waste disposal tends to be mixed as a whole. The utilization level highlights the high utilization level of waste produced in the industrial field; however, in terms of recycling technology, there is a lack of professional technology in the industry, agriculture, or other fields, and most belong to the mixed field. To sum up, China’s solid waste resource recycling policy has some problems in different industries, such as uneven distribution, unbalanced implementation, input–output mismatch, and so on [28,29].

### 3.2. Literature Research and Analysis under the Dimension of Technical Characteristics

**Figure 5:**
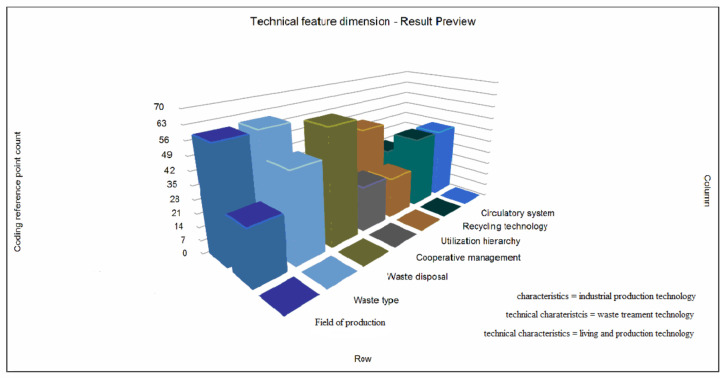
Counting diagram of matrix coding reference points under the dimension of technical characteristics.

In the dimension of technical characteristics, according to the thought map set up in the early stage, seven aspects of solid waste resources in China are selected from the secondary nodes under the initial generation, existing management, and recycling of the NVivo primary node, including “generation field”, “waste type”, “waste disposal”, “cooperative management”, “utilization level”, “recycling technology”, and “recycling system”, and generate the matrix coding reference point counting chart. As can be seen from Figure 5, on the basis of existing literature research, industrial production technology and waste treatment technology participate more in the recycling of solid waste resources, while domestic production technology has a lower participation. Some scholars conducted research and analysis on the problem of solid waste in China’s ceramic industry from a technical point of view, emphasizing that “the treatment and utilization of solid waste in ceramic industry has become a common concern of ceramic workers and environmental engineering technicians” [9]. It can be seen from the 3D histogram that industrial production technology has obvious advantages in the field of solid waste generation, types of production waste, and utilization levels, which represents the high participation of industrial production technology in these aspects. Waste treatment technology plays a greater role in waste disposal, recycling technology and recycling systems. It must be pointed out that, in terms of cooperative management of solid waste recycling, the participation of technology is not high in general, and only a small proportion of waste treatments in technology is involved. Secondly, there are obvious differences between utilization levels, recycling technology, and recycling systems, indicating that industrial production technology has not been fully developed in the process of participating in recycling. Therefore, combined with the above analysis, China’s solid waste resource recycling policy should strengthen integration with technology, promote the implementation of the policy with technology, and pay attention to the intersection and cooperation between different technologies, so as to truly benefit from the recycling of technology [30,31].

### 3.3. Literature Research and Analysis under the Dimension of Policy Attribute

**Figure 6:**
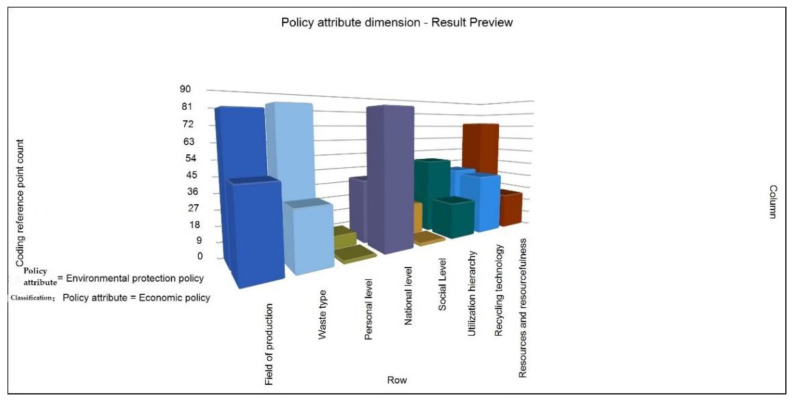
Counting Chart Of Matrix Coding Reference Points Under Policy Attribute Dimension.

Through the reading and division of the literature, the policy attributes are specifically divided into two categories: environmental protection policy and economic policy. The technical diagram of the matrix coding reference points is formed with eight secondary nodes, “generation field”, “waste type”, “personal level”, “national level”, “social level”, “utilization level”, “recycling technology”, and “resources and recycling” (see Figure 6). It can be seen from the figure that environmental protection policies have a higher impact on the field of waste generation and types of waste, which leads to a higher level of utilization, resources, and recycling level. In terms of the overall correlation between the national, social, and individual levels and environmental protection policies and economic policies, the participation at the national level has absolute advantages, among which the economic policy is the highest level. On the contrary, at the individual and social levels, the current situation is more exposed to environmental protection policies; recycling technology is roughly at the same level. Some researchers have also pointed out that “We should give full play to the role of economic incentive policies and social policies in promoting the development of resource recycling industry and optimize the combination of policy tools [10]. On the one hand, we should strengthen the direct correlation between resource recycling and the interests of residents and enterprises through economic incentive policies, stimulate price sensitive consumers and cost sensitive enterprises to actively recycle resources, and strengthen the recycling of development resources from the two aspects of fiscal, taxation and financial policies Support of environmental utilization; On the other hand, we should enrich the contents and forms of social policies and strengthen education on resource conservation and environmental protection, green lifestyle and green consumption”. Based on the above considerations, for the recycling of solid waste resources, the policy attributes should be reasonably matched to ensure that the dual policy ability and cooperation ability of economic policy and environmental protection policy can be fully mobilized, so as to give full play to the absolute advantage of policy governance [32,33].

### 3.4. Literature Research and Analysis under the Dimension of Circular Background

**Figure 7:**
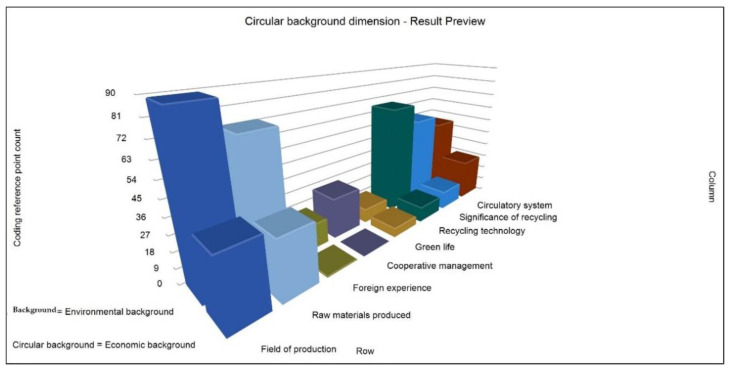
Reference point counting diagram of matrix coding under cyclic background dimension.

The so-called recycling background dimension mainly refers to the main reasons for the solid waste resource recycling policy. It is divided into two subdimensions: environmental protection background and economic benefit background. At the same time, Figure 7 is generated with the secondary nodes “production field”, “production raw materials”, “foreign experience”, “cooperative management”, “green life”, “recycling technology”, “recycling significance”, and “recycling system”. Generally speaking, the environmental background plays a greater role in the recycling policy of solid waste resources, that is, environmental protection requirements are the main reason for the recycling policy of solid waste resources in China, followed by economic factors. Some scholars have pointed out that “China’s economic and social development under the constraints of resources and environment, reducing resource consumption and improving resource utilization is one of the important strategies to realize the construction of ecological civilization [11]. Exploring the coupling relationship between resource recycling and ecological civilization construction is of great significance to environmental governance and green development in the life cycle of resources”. It can also be seen from the figure that China’s reference to foreign experience in this regard is still limited, and the level of cooperative management is also at a low level. Therefore, the integration of the environmental background and economic background is inevitable, which needs further reference, research, and application. On the premise of taking into account the environmental and economic reasons for the policy, we can provide the correct direction for various policy activities in the later stages [34,35].

## 4. Research Literature Emotional State

**Figure 8:**
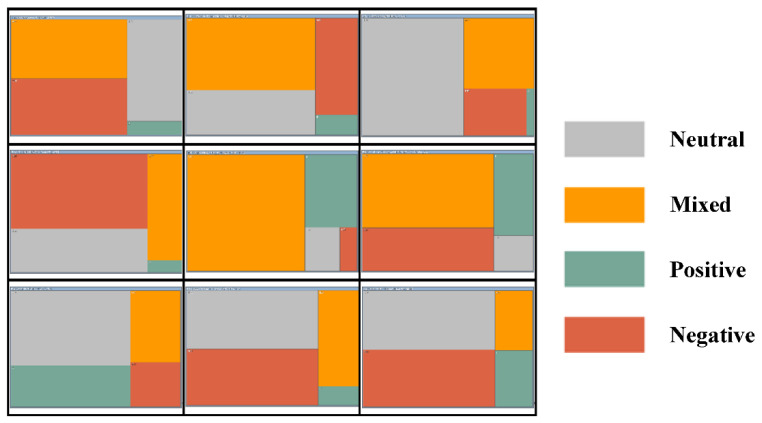
Automatic coding of emotional results hierarchy chart.

Through the screening of ten selected studies and the automatic coding recognition emotion analysis by NVivo software, a statistical table of coding points and a summary diagram of the hierarchical chart of nine studies compiled according to emotion are obtained. According to the statistics in Table 1, “very negative” has 259 coding nodes in total; “Relatively negative” has 69 coding nodes in total; there are 205 coding nodes for “relatively negative direction”; “Very positive” has 10 coding nodes in total. As can be seen in Figure 8, “neutral” and “mixed” attitudes occupy absolute advantages. Therefore, based on the results of Table 1 and Figure 8, Chinese scholars’ attitudes towards the recycling policy of solid waste resources is still in a complex period, though they tend to show that, although the existing relevant policies have achieved certain results, they still have great room for development and prospects.

## 5. Conclusions

With the help of NVivo 11, this study undertakes a text analysis of ten articles in Peking University core and CSSCI journals derived from China HowNet. Through the construction of a mind map, word frequency cloud map and matrix coding, this paper studies the recycling policy of solid waste resources in China, analyzes its qualitative, emotional, and other contents, and obtains research results. The following are some shortcomings of this research and the future prospects of this research problem.

### 5.1. Research Deficiencies

Firstly, the literature selected in this study is from the core of Peking University and CSSCI literature derived from CNKI, with a total of 10. Regarding its use in NVivo software, this number of studies has no research advantage. Secondly, this study did not select a specific policy on the recycling of solid waste resources in China, therefore there is a lack of example policy text analysis in the research content. Finally, it should be highlighted that there are still many unskilled aspects in the application of NVivo software, which may lead to the failure of some research.

### 5.2. Future Prospects of Recycling Policies of Solid Waste Resources in China

#### 5.2.1. Strengthen Planning and Build an Integrated Policy System

In the face of the significant differences in the allocation, implementation, and effect of policies in different industries, it is necessary to clarify the causes of the problems, that is, the lack of integrated planning and design. Therefore, it is necessary to strengthen the leadership of the upper level and coordinate and guide China’s solid waste resource recycling policies from a strategic perspective. Full play should be given to the mandatory role of laws and regulations, and legal procedures for the policy process should be established, as should a special policy implementation control department to ensure the overall legitimacy and standardization of the policy process. At the same time, we should also further supervise the formulation of policies to achieve balance between pertinence and universality, coordination, and restraint. In addition, we should strengthen the standardization of policy methods, standardize the implementation of policies with standardization, and ensure a high level of policy output effects such as economic and ecological benefits [36].

#### 5.2.2. Actively Cooperate and Enhance Technical Policy Capacity

In the face of the problem of recycling of solid waste resources in China, the technical achievements behind it must have high requirements. Therefore, as far as policy tools are concerned, we should support and help with the integration of technology applications, focus on the importance of building a technologically practical platform, promote the participation of diversified technology industries, and improve the comprehensive ability and level of recycling of solid waste resources in China. In addition to this technical requirement, we should also improve and enrich the technology of think tanks behind the solid waste resource recycling policy, mainly focusing on their network technology and the professional quality of think tank personnel, improving the group voice and professionalism of policymakers so as to facilitate the tracking, analysis, and discussion of the real-time environment at home and abroad, and strengthen the contact and communication with various governmental scientific research institutions, social enterprises, and the public. This will promote the orderly development of China’s solid waste resource recycling industry [37,38].

#### 5.2.3. Make Reasonable Arrangements and Improve Diversified Policies and Measures

Facing the trade-off between the two policy attributes of economic policy and environmental protection policy, the conclusion is to give full play to their advantages, try to avoid their disadvantages, and realize the organic combination of the two policy tools. On the one hand, we should use the economic attribute to strengthen the efficient integration of China’s solid waste resource recycling and social capital, attract highly relevant consumers and producers, and actively participate in the recycling of solid waste resources from the two dimensions of consumption level and production cost, which can further deepen the incentive role of fiscal policy. On the other hand, we should enrich the contents and forms of environmental protection policies, actively develop various methods of publicity and education, publicize various forms of knowledge conducive to the promotion of waste resource recycling, such as resource conservation, environmental protection, ecological economy, and green life, and realize the social atmosphere of all relevant subjects of society actively participating in waste resource recycling as soon as possible [38].

#### 5.2.4. Adapt to Internal and External Factors, Coordinate, and Refine the Policy Environment

In the face of the increasingly complex social environment, the dual influence of environmental protection background and economic background, as well as the inevitable trend of comprehensive exchange and development at home and abroad, China’s solid waste resource recycling work should also realize industrialization development. In the face of the goal of recycling industrialization, policy formulation and implementation should actively change the traditional form, carry out the “transformation and upgrading” of the policy itself, and learn from the advanced experiences at home and abroad, finely distinguishing and taking root in the specific environmental background. Policymakers should seize the key time point of the development of the recycling industry, strengthen targeted policy output, and strive to create a regulatory environment, operating environment, and exchange environment conducive to the development of the recycling industry and the implementation of relevant policies, so as to realize the fine integration of policies and the waste resource recycling industry. We should also pay extra attention to policy support for cooperation and exchange with foreign countries and introduce China’s solid waste resources to the international market with the help of the “the Belt and Road” international cooperation channel, so as to achieve “domestic and international double circulation” [39,40].

The raw data supporting the conclusions of this manuscript will be made available by the authors, without undue reservation, to any qualified researcher.

## Figures and Tables

**Table 1:** Summary of automatic coding of emotional results.

Entry	Literature Name	Very Negative	More Negative	More Positive	Very Positive
1	Mineralogy of recycling of industrial solid waste resources	54	25	42	3
2	Research on classification and resource utilization of solid waste based on life cycle management	42	5	24	0
3	Current situation of industrial utilization of solid ceramic waste	14	9	14	0
4	Application of input-output analysis in solid waste management	34	3	14	0
5	Promote the comprehensive utilization of bulk solid waste Promote the development of resource recycling industry	12	1	11	0
6	China and the “the Belt and Road” jointly build countries Current situation of waste management and suggestions for cooperation	49	3	43	1
7	Research on the standardization of resource recycling in China	11	10	13	3
8	Study on the theoretical connotation and system model of resource recycling in China	17	2	12	0
9	Study on the evolution characteristics of industrial policy of resource recycling in China	26	11	32	3

## Data Availability

The data used to support the findings of this study are available from the corresponding author upon request.
